# Supplementary material for: Potential classification of chemical immunologic response based on gene expression profiles
Source: J Immunotoxicol. Author manuscript; Available in PMC 2020 Dec 1. (PMC7673648; doi:10.1080/1547691X.2020.1758855)
Supplement: final draft w supplementary figures [file NIHMS1639316-supplement-final_draft_w_supplementary_figures.doc]

**Potential Classification of Chemical Immunologic Response Based on Gene Expression Profiles**

Stacey E. Anderson, Rachel Baur, Michael Kashon, Ewa Lukomska, Lisa Weatherly, and Hillary L. Shane

Allergy and Clinical Immunology Branch, Health Effects Laboratory Division, National Institute for Occupational Safety and Health, Morgantown, WV. 26505

Please address all correspondence to: Stacey Anderson, PhD, National Institute for Occupational Safety and Health (NIOSH), 1095 Willowdale Drive, Morgantown, WV 26505. (T) 304-285-6024 (F) 304-285-6126 (E) sanderson4@cdc.gov

**Running Title:** Antimicrobial Chemical Gene Expression Profile

**Keywords:** Immune, antimicrobials, skin, gene expression

**Abstract**

Occupational immune diseases are a serious public health burden and are often a result of exposure to low molecular weight (LMW) chemicals. The complete immunological mechanisms driving these responses are not fully understood which has made the classification of chemical allergens difficult. Antimicrobials are a large group of immunologically-diverse LMW agents. In these studies, mice were dermally exposed to representative antimicrobial chemicals (sensitizers: didecyldimethylammonium chloride (DDAC), *ortho*-phthalaldehyde (OPA), irritants: benzal-konium chloride (BAC), and adjuvant: triclosan (TCS)) and the mRNA expression of cytokines and cellular mediators was evaluated using real-time qPCR in various tissues over a 7-d period. All antimicrobials caused increases in the mRNA expression of the danger signals *Tslp* (skin), and *S100a8* (skin, blood, lung). Expression of the T_H_2 cytokine *Il4* peaked at different timepoints for the chemicals based on exposure duration. Unique expression profiles were identified for OPA (*Il10* in lymph node, *Il4* and *Il13* in lung) and TCS (*Tlr4* in skin). Additionally, all chemicals except OPA induced decreased expression of the cellular adhesion molecule *Ecad*. Overall, the results from these studies suggest that unique gene expression profiles are implicated following dermal exposure to various antimicrobial agents, warranting the need for additional studies. In order to advance the development of preventative and therapeutic strategies to combat immunological disease, underlying mechanisms of antimicrobial-induced immunomodulation must be fully understood. This understanding will aid in the development of more effective methods to screen for chemical toxicity, and may potentially lead to more effective treatment strategies for those suffering from immune diseases.

**Introduction**

Millions of workers have the potential for dermal and/or respiratory exposure to low molecular weight (LMW) chemicals which can result in occupational diseases. While the number of chemicals used in industrial applications now exceeds 82,000, according to the EPA, ≈ 3000 additional new chemicals are introduced every year. Workplace exposures can result in a number of allergic diseases; ≈ 11 million American workers are at risk for exposure to agents that can cause allergic disease (Anderson and Meade 2014a). Exposures to certain LMW chemicals can impact immune function that can result in uncontrolled inflammation, increased susceptibility to infection and disease, or allergic disease. These conditions may be detrimental to a worker’s health and workplace performance, causing significant economic losses (Cashman et al. 2012).

Allergic conditions are exaggerated immune responses, resulting in disease outcomes including asthma and allergic contact dermatitis (ACD). These often occur in response to LMW sensitizers found in the workplace. Health experts believe that between 15 and 23 percent of all cases of asthma may be related to working conditions (Pralong et al. 2012; Dotson et al. 2015). Contact dermatitis (irritant and allergic) is also a common chemically-induced occupational disease, accounting for 15-20% of all occupational illnesses, with an estimated annual cost of at least $1 billion (Sasseville 2012). While many chemicals are known to directly induce allergic disease, there is also the potential for non-allergenic chemicals to function as adjuvants or irritants, augmenting the immune responses induced by other chemical and protein allergens.

Antimicrobials represent a broad class of LMW chemicals with the intended purpose of eliminating or controlling the growth of harmful microorganisms. Exposure to these agents can occur occupationally or via use/consumption of consumer products. The use of antimicrobial agents has been associated with an increased incidence of allergic diseases, including asthma, atopic dermatitis, and less commonly, anaphylaxis. Very diverse immunological mechanisms and mediators have been identified in the sensitization response to antimicrobials (Anderson et al. 2019). Specifically, common antimicrobials *o*-phthalaldehyde (OPA), benzalkonium chloride (BAC), didecyldimethylammonium chloride (DDAC), and triclosan (TCS) have been associated with immunological diseases (Anderson and Meade 2014b; Anderson et al. 2019). Quaternary ammonium compounds (QAC) are a specific class of antimicrobials (used in sprays and wet-wipe products used to disinfect surfaces and floors) and are recognized as common occupational allergens due to their association with both contact dermatitis and occupational asthma (Bernstein et al., 1994; Shaffer and Belsito 2000; Suneja and Belsito, 2008; Gonzalez et al., 2014; Anderson et al., 2016b; Shane et al., 2017; Shutty and Scheinman, 2017). The QAC BAC is commonly associated with asthma in humans; however, it is typically classified as an irritant (or weak sensitizer) in animal studies (Manetz and Meade 1999). DDAC, another QAC, is a broad-spectrum bactericidal and fungicidal biocide (Skaliy et al. 1980). Allergic contact dermatitis and immediate-type allergic reactions caused by DDAC exposure have been reported (Dejobert et al., 1997; Dibo and Brasch, 2001; Ruiz Oropeza et al. 2011; Mowitz and Ponten, 2015). In animal models, DDAC has been identified as an irritant and strong T-cell-mediated sensitizer based on cellular phenotyping and the lack of identification of serum IgE (Anderson et al. 2016b). OPA is an aromatic dialdehyde used as a high-level antimicrobial disinfectant for medical equipment which is sensitive to normal heat or steam sterilization processes. Exposure has been associated with anaphylaxis, occupational asthma, and severe allergic reactions in humans (Sokol, 2004; Fujita et al. 2007). Additionally, animal studies have identified OPA as an IgE-mediated sensitizer (Anderson et al., 2010).

While certain antimicrobials - including those described above - are known to induce sensitization, others such as TCS have been associated with allergic disease, though not directly sensitizing. In addition to its clinical use, TCS is used as a preservative, fungicide, and biocide in household and personal care products (Glaser, 2004; Fang et al., 2010; Weatherly and Gosse, 2017). Research suggests that TCS exposure may be at least in part responsible for recent increases in the frequency of asthma and allergic disease (Savage et al., 2012, 2014; Anderson et al. 2013). Additional studies have revealed that topical exposure to TCS augmented the allergic response to an experimental allergen through a thymic stromal lymphopoietin (TSLP)-mediated signaling pathway in a mouse model of asthma (Anderson et al. 2013; Marshall et al. 2015).

Exposure to antimicrobial chemicals can result in multiple hypersensitivity pathways/ disease outcomes (i.e. both IgE-mediated/T-cell-mediated; asthma/allergic contact dermatitis), reflecting an increased complexity of immunological mechanisms driving these response. Further research is needed to evaluate the hazard-potential associated with antimicrobials and to fully understand the immunologic mechanisms that induce and exacerbate immune and allergic diseases. Moreover, identification of specific biomarkers would help to identify potential immune responses resulting from exposure. Ultimately, a complete understanding of mecha-nisms of allergic diseases resulting from antimicrobial exposure will allow for surveillance, proper treatment and/or prevention, while hazard identification will lead to risk assessment, which will ensure safe environments and exposure limits.

In the studies described here, gene expression profiles were examined using real-time qPCR following dermal exposure to the above-mentioned antimicrobial chemicals to identify unique profiles that could potentially aid in hazard classification and provide a better understand-ing of mechanisms involved. Based on human and animals study findings, the antimicrobials used were a weak sensitizer/irritant (BAC), IgE-mediated sensitizer (OPA), T-cell-mediated sensitizer (DDAC), or an adjuvant (TCS). Expression of cytokines and cellular mediators were then analyzed in mouse skin, draining lymph nodes (dLN), blood, and lungs after repeated chemical exposures. It is hoped the findings here will contribute to a more complete understand-ing of mechanisms of immune diseases resulting from antimicrobial exposure, and will help to ensure safe workplace environments and effective exposure limits.

**Materials and Methods**

**Animals**

BALB/c mice (female, 7-8-wk-old) were purchased from Taconic (Germantown, NY). Upon arrival, mice were allowed to acclimate for a minimum of 5 d. Each shipment of mice was randomly assigned to an exposure group and identified with tail markings made by a permanent marker. Mice were housed (five/cage) in ventilated plastic shoebox cages with hardwood chip bedding. Harlan NIH-31 modified 6% irradiated rodent diet and filtered tap water were available *ad libitum*. Housing facilities were maintained at 68-72°F and at a 36-57% relative humidity, with a 12 hr light/dark cycle. All animal experiments were performed in the AAALAC International accredited National Institute for Occupational Safety and Health (NIOSH) animal facility in accordance with an animal protocol approved by the CDC-Morgantown Institutional Animal Care and Use Committee (IACUC).

**Test chemicals**

Benzalkonium chloride (BAC, CAS# 63449-41-2) and *o*-phthalaldehyde (OPA, CAS# 643-79-8) were purchased from Sigma (St. Louis, MO). Didecyldimethylammonium chloride (DDAC, CAS# 7173-51-5) was purchased from AKSci (Union City, CA). Triclosan (TCS, CAS# 3380-34-5) was purchased from Calbiochem (Burlington, MA). Acetone (CAS# 67-41-1) was purchased from Acros (Waltham, MA).

**Chemical exposures**

Mice (five/group) were exposed once per day for either 1, 2, 4, or 7 consecutive days to vehicle (acetone) or to one of three concentrations of test chemical (BAC 0.5%, 1%, 2%; OPA 0.25%, 0.5%, 0.75%; DDAC 0.125%, 0.25%, 0.5%; TCS 0.75%, 1.5%, 3%) on the dorsal surface of each ear (25 µl/ear). Concentrations were selected based on previous study findings (Anderson et al. 2010, 2016a,b). Acetone was selected as the vehicle based on solubility and previous use in evaluations of chemical sensitization (Table 1). Expression of cytokines and cellular mediators was analyzed in the mouse skin, dLN, blood, and lung 24 hr after the final exposure (see below). Antimicrobials were selected based on their classification as irritant, IgE mediated sensitizer, T-cell-mediated sensitizer, or adjuvant (Table 1). For the sensitizing chemicals, an irritating/sensitizing and non-irritating/sensitizing concentration were included.

**Euthanasia and tissue collection**

Animals were euthanized by CO_2_ inhalation 24 hr after the final exposure. Left and right ears, left and right auricular dLN, and lung were collected into tubes containing 500 µl RNAlater (Invitrogen, Carlsbad, CA)). Blood was collected from the abdominal aorta and placed into tubes containing 700 µL QIAzol Lysis Reagent (Qiagen, Germantown, MD)). Samples were frozen at -80°C until processed.

**RNA isolation and reverse transcription**

Total RNA was isolated from the ear (RNeasy kit), dLN (miRNeasy kit), blood (miRNeasy kit), and lung (miRNeasy kit for OPA and DDAC; RNeasy kit for BAC and TCS) according to manufacturer protocols (Qiagen). A QIAcube (Qiagen) automated RNA isolation machine was used in conjunction with the specified RNA isolation kit. The concentration and purity of the isolated RNA was determined using a NanoDrop Spectrophotometer (Thermo Scientific, Waltham, MA)). Reverse transcription was performed using a High-Capacity cDNA Reverse Transcription Kit (Applied Biosystems, Foster City, CA) according to manufacturer recom-mendations.

**Gene Expression Analysis**

TaqMan Fast Universal PCR Master Mix (Applied Biosystems), cDNA, and gene-specific primers (TaqMan Gene Expression Assays) were combined and real-time quantitative PCR was performed according to the manufacturer’s directions. Genes tested include: *S100a8, Tslp, Il13, Il33, Il4, Tnip1, Tnfaip3* (lung); S100a8, *Rage*, *Tnip1*, *Tnfaip3 (blood)*; *Tslp*, *Foxp3*, *Cdh1*, *Tlr4*, *Il4*, *Il13*, *Il22*, *(ear)*, and *Ifng, Il-4, Il-5, Il10, Foxp3 (dLN)*. *Actb* was used as the reference gene. Genes were selected based on known or suspected immunological roles in the specified tissue. MicroAmp Fast Optical 96-Well Reaction Plates were analyzed in a 7500 Fast Real-Time PCR System (Applied Biosystems) according to manufacturer directions. Data was collected and represented as the relative fold-change compared to vehicle control using the cycle threshold (C_t_) and the 2^-ΔΔCt^ method.

**Statistical analysis**

The PCR data generated from these experiments were analyzed using SAS/STAT for Windows (v9.4) and JMP for Windows (v13). For each chemical utilized in these studies, two-way (Concentration by Day) analysis of variance was performed for each molecule using Proc Mixed in SAS. Data derived using the 2^-ΔΔCt^ method were log-transformed prior to analysis to meet the assumption of homogeneous variance for the statistical model. Significant differences across days, and concentrations were assessed using Fishers LSD test. All differences were considered significant at p < 0.05. Heat maps were generated using JMP version 13.

**Results**

**Antimicrobial chemical exposure increases danger signal expression**

Several factors and molecular signals play a role in whether chemical exposure leads to sensitization. The two signals that are essential in order for a chemical to result in sensitization are T-cell activation and the presence of danger signals (Shane et al. 2019). Thymic stromal lymphopoietin (TSLP) is a danger signal typically associated with the activation of Type 2 helper T-cell (T_H_2) responses. Previous work in our laboratory has shown that TCS augments the allergic response through a TSLP-mediated pathway (Marshall et al. 2015).

Dermal exposure to all of the antimicrobial chemicals resulted in a significant increase in *Tslp* mRNA expression at the site of exposure (Figure 1A). DDAC and TCS exposure led to a peak of *Tslp* expression (580 and 35 fold, respectively) after 4 days, whereas BAC and OPA exposure led to a peak (180 and 80 fold, respectively) of *Tslp* expression after just 2 d of exposure. For the sensitizing chemicals (DDAC and OPA), both irritating/sensitizing and non-irritating/sensitizing concentrations induced significant changes in *Tslp* expression. Another danger signal, S100A8, has also been identified as a factor in the adjuvant effect of TCS exposure (Marshall et al. 2017). S100A8 forms a heterodimer with S100A9, resulting in the danger signal protein called calprotectin. Dermal exposure to all antimicrobial chemicals resulted in an increase in *S100a8* mRNA expression (Figure 1B). However, unlike *Tslp* expression, *S100a8* expression continued to increase during the 7 d of exposure for all concentrations of the tested chemicals. For the sensitizing chemicals (DDAC and OPA), both irritating/sensitizing and non-irritating/sensitizing concentrations induced significant changes in *S100a8* expression.

Dermal exposure to chemicals has systemic effects, including enhancing allergic responses in the lungs; thus, expression of danger signals in the lungs of mice following test agent exposure were evaluated. Dermal exposure to the antimicrobial chemicals here did not alter expression of *Tslp* in the lungs over 2-fold (data not shown). Interestingly, the exposures did increase *S100a8* expression in the lungs (Figure 2A). Dermal exposure to BAC led to a statistically significant increase in *S100a8* expression at mid and high concentrations at all days (Figure 2B). Exposure to the highest TCS concentration significantly increased *S100a8* expres-sion in the lungs at all days and just 1 d of TCS exposure increased *S100a8* at all concentrations at that site. Exposure to DDAC for 1, 2, 4, and 7 d increased *S100a8* expression in the lungs at the highest concentration, and at the mid concentration after 4 d of exposure. Similar responses were observed in the blood for all chemicals evaluated (Figure 2B). Blood following 7 d of DDAC exposure was not evaluated due to equipment failure.

**OPA exposure increases TH2 cytokine levels**

Interleukin (IL)-4 is a central cytokine in T_H_2 immune responses. Expression of *Il4* mRNA was assessed in the skin, dLN, and lungs following dermal exposure to antimicrobial chemicals. OPA exposure significantly increased *Il4* mRNA expression in the skin at the low, mid, and high concentrations after 4 and 7 d of exposure, in the skin at the mid and high concentrations after 2 d of exposure, and in the skin at the high concentration after just 1 d of exposure (Figure 3A). Seven days of BAC or DDAC exposure at their highest concentrations led to significant increases in *Il4* in the skin. Exposure to the highest concentration of TCS for 2, 4, and 7 d also led to statistically significant increases of *Il4* in the skin. *Il4* was increased in the dLN following 2 d of the highest OPA exposure and following 4 and 7 d of all tested OPA levels (Figure 3B). Seven days of TCS exposure at all concentrations resulted in a significant increase in *Il4* in the dLN and 4 d of the highest concentration of TCS led to a significant increase in *Il4*. Exposure to BAC or DDAC for 7 d resulted in statistically significant increases in *Il4* expression at all test concentrations.

Dermal exposure to OPA for 7 d increased *Il4* expression in the lungs at all test levels (Figure 4A). IL-13 is another cytokine central to the T_H_2 immune response. Dermal exposure to OPA for 7 d also increased *Il13* expression in the lungs at all test concentrations (Figure 4B). None of the other antimicrobials altered expression of *Il4* or *Il13* in the lung (data not shown).

**OPA exposure influences the regulatory response**

Regulatory T-cells (T_reg_) have previously been found to be involved in the immune response to chemical exposure (Long et al. 2016). FoxP3, the critical transcription factor for T_reg_ cell development was assessed in the skin following the chemical exposures. Dermal exposure to BAC, OPA, and DDAC for 7 d increased *Foxp3* expression in skin at all tested concentrations (Figure 5A). Exposure to TCS for 7 d at the highest concentration also increased the expression. OPA exposure also significantly increased *Foxp3* expression after 4 d of all test concentrations and after 2 d of the mid and high concentrations. Similar, but less dramatic results, were obtained in the dLN (Supplemental Figure 3). Expression of *Il10*, a cytokine produced by T_reg_ cells and involved in immune regulation, was assessed in the dLN following dermal chemical exposure. OPA exposure significantly increased *Il10* expression in the dLN after 7 d of all test levels and after 4 d of exposure to the highest concentration (Figure 5B). Exposure to DDAC for 2 d significantly decreased *Il10* expression in the LN at the mid and high concentrations.

**Exposure to TCS uniquely alters *Tlr4* expression in skin**

Understanding the mediators involved in chemical sensitization and immune responses after dermal chemical exposure is critical in identifying the differences between sensitizers, irritants, and adjuvants. Toll-like receptor 4 (TLR4) has previously been identified to play a role in the immune response to TCS (Marshall et al. 2017). *Tlr4* expression was therefore assessed in the skin following the dermal chemical exposures. Exposure to 7 d of TCS at the highest concentration increased *Tlr4* expression in the skin (Figure 6A). However, no other chemical significantly increased this expression.

**Decreases in E-cadherin expression after antimicrobial exposures**

E-cadherin, a cellular adhesion molecule highly expressed in the skin and associated with innate lymphoid cells (ILC), has been shown to suppress T_H_2 cytokine production by Type 2 innate lymphoid cells (ILC2) through ligation with the co-inhibitory receptor killer-cell lectin like receptor G1 (KLRG1). E-cadherin is also associated with inflammatory skin diseases such as atopic dermatitis (Salimi et al. 2013). Exposure for 4 and 7 d to TCS and DDAC at the highest concentration decreased E-cadherin (*Cdh1*) expression in the skin (Figure 6B). *Cdh1* expression was also decreased following exposure to the high concentration of BAC at Day 7 and the mid concentration at Days 2, 4, and 7. OPA exposure did not alter *Cdh1* expression.

**Chemical exposure increases *Ifnγ* and *Il22* expression**

Interferon (IFN)-γ is the cytokine central to Type 1 helper T-cell (T_H_1) responses. OPA exposure significantly increased *Ifnγ* mRNA expression in the dLN after just 2 and 4 d of exposure at all test concentrations, and after 1 d of exposure to the highest concentration (Figure 7A). Exposure to BAC significantly increased *Ifng* expression after 1 d of exposure to the high concentration. Interestingly, expression was significantly decreased (mid and high levels) after 7 d of exposure. Exposure to DDAC significantly decreased *Ifng* expression for all concentrations 2 d post-exposure; this decrease persisted until 7 d post-exposure. No changes in *Ifnγ* expression were seen following exposure to TCS.

IL-22 is a cytokine expressed by Type 17 helper T-cells (T_H_17). *Il22* mRNA expression was assessed in the skin following dermal exposure to chemicals. All chemicals resulted in significant increases in *Il22* expression after exposure. Exposure to TCS for 4 or 7 d significantly increased *Il22* expression at all concentrations, with a peak increase after 4 d of exposure (Figure 7B). Dermal exposure to BAC or DDAC increased *Il22* expression after 4 and 7 d, but the peak increase occurred after 7 d of exposure (Figure 7B). OPA significantly increased expression of *Il22* by 1 d post-exposure, with a peak increase at 4 d that persisted until 7 d post-exposure.

**Discussion**

Occupational immune diseases are a serious health burden. Thus, the ability to identify chemical hazards and understand immunological mechanisms of disease is critical. Numerous studies have shown that exposure to chemicals can drive the development of allergic diseases, either directly, or indirectly. The results from this study identify unique expression profiles of select cytokines and cellular mediators between different classes of antimicrobial chemicals (Supplemental Figures 1-4). For this study, the representative chemicals were classified based on a specific type of immune response (Table 1). It is important to note that this classification scheme represents a simplified approach, as the categorization of immune responses is more complex than what is presented. The wide spectrum of clinical symptoms associated with most of the investigated chemicals suggest mixed immune responses and hypersensitivity classifications that may not be mutually exclusive. However, in the studies described here, irritating/sensitizing and non-irritating/sensitizing concentrations were evaluated for the sensitizing chemicals. Although direct comparisons may be difficult to make due to differences in exposure concentration and potency, unique chemical trends can still be identified.

For this study, OPA was classified as an irritant and IgE-mediated sensitizer based on findings in previous human and animal studies. Studies conducted in our laboratory found that exposure to OPA significantly increased ear swelling and lymphocyte proliferation in the dLN when evaluated in the local lymph node assay (LLNA) (Anderson et al. 2010). In addition, 0.5% OPA exposure increased serum IgE along with IL-4 expression at both the gene and protein level in the dLN . Consistent with these findings which further support its T_H_2 classification, OPA was identified to induce early and persistent expression of *Il4*, following dermal exposure to multiple concentrations, in the ear and dLN. Interestingly and uniquely, OPA also induced expression of *Il4* and *Il13* in the lungs. While involvement of the skin is recognized in the development of dermal sensitization, it has recently been implicated in the development of systemic sensitization leading to elicitation responses at various sites in the body, including the respiratory tract (Bello et al. 2007). This has been demonstrated in animal studies involving both protein and chemical allergens (Zhang et al., 2002 ;Herrick et al. 2003; Redlich 2010). In a workplace setting, the like-lihood of dermal contact with low molecular weight (LMW) chemicals is high, further support-ing the idea that dermal exposures may lead to respiratory allergic disease. In addition to their probability of occurrence in the workplace, LMW chemical skin exposures also have a potential for higher dose-delivery in comparison to inhalation exposures (Bello et al. 2007). These findings support a very interesting connection and potential discriminating feature between respiratory and contact sensitizers and further suggests that sensitization via the skin may be important for respiratory allergic outcomes.

The development of chemical allergy is immunologically complex and our understanding of the mechanisms driving these responses continue to evolve. Research suggests that dosage, exposure duration, and route of exposure may all influence/alter a developing immune response. Adding to the complexity of defining immune responses is the increased understanding that the development of hypersensitivity responses is not as divergent nor categorical as once thought. It is generally accepted that sensitizing chemicals that induce T_H_2 and/or IgE-mediated responses will tend to initiate expression of T_H_2 cytokines and suppress those commonly associated with T_H_1 effector responses (Dearman and Kimber 1991; Kimber and Dearman, 1992; Anderson et al., 2011). Despite this, the specific chemical properties that define each type of sensitizer have not been identified.

In contrast to OPA, DDAC has been identified as an irritant and strong T-cell-mediated sensitizer in mice. Exposure to 0.5% DDAC was previously shown to result in increased ear swelling with a significant increase in lymphocyte proliferation at 0.25%, when evaluated in the LLNA but did not increase serum IgE levels (Anderson et al. 2016b). Although classified as a T-cell sensitizer in these studies, here, DDAC induced significant expression of *Il4* in the skin and dLN following 7 d of exposure. This is consistent with increases previously identified in IL-4 expression following DDAC exposure at both the transcript and protein level (Shane et al. 2019b). It is possible that this early IL-4 production is due to innate mediators such as ILC2, and may contribute to a mixed-type response. Additional studies conducted in our laboratory have also demonstrate that extended dermal exposure to QAC (14 days+) induced production of serum and local IgE (Shane et al. 2017, 2019b). Interestingly, significant decreases in mRNA expression of the T_H_1 cytokine *Ifng* that persisted throughout the course of the study, were identified following DDAC exposure. In contrast, OPA significantly increased expression of *Ifng*. BAC was included in the present study as an irritant/weak sensitizer based on findings in human and animal studies (Manetz and Meade 1999; Isaac and Scheinman, 2017). For BAC, an immediate increase in *Ifng* was observed, but this did not persist after the 1 d timepoint. These findings further demonstrate the induction of mixed responses by LMW chemicals and support the impact of exposure duration on the subsequent immunological response.

Although chemicals can directly affect the immune system and subsequently influence allergic disease, there is also the potential for indirect affects through mechanisms involving irritation/inflammation and adjuvancy. While associated with allergic disease in humans, *in vivo* hazard identification models have not identified TCS as a sensitizer or irritant (Anderson et al. 2016a). However, dermal TCS exposure has been shown to augment the allergic response to an experimental allergen in a mouse model of asthma; thus, TCS was classified as an adjuvant for this study (Anderson et al., 2013, 2016a). While TCS exposure induced expression profiles that were similar to the sensitizers (*Tslp, S1000a8, Il22, Il4, Foxp3, Cdh1*), unique to exposure were increases in expression of *Tlr4* in the skin. A similar finding has been previously described in our laboratory (Marshall et al., 2017). More specifically, in the current and previous studies, TCS was seen to induce abundant expression of *S100A8* in the skin; this protein acts as an endogenous ligand for the intracellular signaling receptor TLR4, which is important for activation of the innate immune system.

The skin serves as a protective layer for our bodies from the outside environment. As the largest organ in the body, the skin is an extremely important player in relation to allergic disease. The presence of multiple innate immune factors including leukocytes, complement factors, antimicrobial peptides, and pattern recognition receptors allow the skin to be a site of immune surveillance and tolerance yet these factors may also contribute to the development of allergic disease (Bangert et al. 2011). The initiation of sensitization begins with exposure and antigen recognition. In order to gain access to immune cells responsible for commencing sensitization, allergens must penetrate the epithelium. In the skin, LMW chemical allergens may be absorbed through the stratum corneum, hair follicles, and sebaceous glands (Nayak et al. 2014), accessing internal cells without physical alteration of the epithelium due to their small size. A widely-accepted concept explaining immunogenicity of LMW chemical allergens involves a haptenation step, i.e., combining with and altering a self-protein, causing an allergic response following presentation by antigen-presenting cells (Landsteiner and Jacobs, 1935; Kohler et al., 1995; Chipinda et al., 2011). Because TCS is not a sensitizing chemical, it does not form a hapten. This lack of reactivity is one potential explanation of why TLR signaling may be unique to this class of chemical.

Recently, the emergence of many novel cellular subsets and molecules involved in immunological responses has occurred, shedding light on unexplored realms of the immune system and their potential involvement in a variety of disease states, including allergic disease (Shane et al. 2019a). In accordance with these developments, further investigation into these responses demonstrated that DDAC induced high levels of expression of the T_H_2-skewing cytokine *Tslp*, which has been shown to activate ILC2 in the skin (Kim et al. 2013). ILC2 are a subset of innate lymphocytes that lack rearranged antigen-specific receptors and produce Type 2 cytokines. ILC2 have recently emerged as important mediators of allergic disease (Cosmi et al. 2017). Following DDAC exposure, ILC2 in the skin were rapidly activated, and their activation coincided with the production of Type 2 cytokines in the absence of T-cells; this provided a potential mechanism for the initiation of the mixed-type allergic response (Shane et al. 2019b). E-cadherin (cellular adhesion molecule highly expressed in skin) has been associated with the suppression of ILC2 function via inhibition of their T_H_2 cytokine production (Salimi et al. 2013). Here, all chemicals except OPA decreased *Cdh1* expression in the skin. However, as numerous signals can drive ILC2 regulation and activation (Dahlgren and Molofsky 2018), it is possible that the ILC2 contribute to early T_H_2 cytokine production in the skin following exposure to OPA.

Another newly characterized helper T-cell subset thought to play a role in allergic disease is the T_H_22 subset. These cells are identified by their production of IL-22 in the absence of IFNγ, IL-4, and IL-17, and are thought to contribute to host defense against microbial pathogens and promote tissue repair or remodeling (Fujita 2013). In the skin, IL-22 plays a major role in home-ostasis and pathogenesis of skin diseases by inducing keratinocyte proliferation and epidermal hyperplasia, inhibiting terminal differentiation of keratinocytes, and promoting the production of antimicrobial proteins. While information about the role of T_H_22 cells in chemical allergy in the skin is lacking, they have been implicated in the pathogenesis of inflammatory skin disorders such as psoriasis and atopic dermatitis (AD) (Mirshafiey et al. 2015). Additionally, IL-22 levels were found to be increased in the skin of patients with AD, ACD, and allergic asthma (Jia and Wu 2014) and IL-22 has been suggested as a potential biomarker for allergic disease (Zissler et al. 2016). In the current study, all chemicals induced *Il22* expression. This outcome supports the need for additional research investigating the role of this cytokine in immunological disease.

Our laboratory has previously shown that chemical sensitizers affect T_reg_ cells (Long et al. 2016). Following single dermal exposure to the known asthmogen toluene diisocyanate (TDI), the LN Treg population expanded significantly at 4, 7, and 9 days. Additionally, Tregs isolated from TDI-sensitized mice were significantly more suppressive compared with their control counterparts, further supporting a functional role for Tregs during sensitization. While here all the tested chemicals induced expression of *Foxp3* in the ear, only OPA increased *Il10* in the dLN. OPA also induced the largest and earliest peak expression of *Foxp3* in the skin. Although the number of T_regs_ was not determined in the present study, the increases in gene expression in addition to our previous findings support a direct role for T_regs_ in chemical sensitization. The collection of data regarding T_reg_ cells and chemical allergy is growing but remains limited. In order to elucidate the immunologic mechanisms involved in LMW chemical sensitization, the biological functions of pertinent immune cell subsets, such as T_reg_ cells need to be delineated.

It has long been recognized that the presence of foreign antigens alone is insufficient to generate immune responses: activation of the innate immune system is also required. Research is continuing to bring to light the importance of such “danger signals” in allergic sensitization. In this study, all chemicals induced expression of the danger signal, *Tslp* in the skin. The highest *Tslp* expression was identified after DDAC exposure, at later timepoints. OPA-induced elevations in *Tslp* expression were generally lower compared to the other sensitizers and had almost returned to baseline by 4 days. However, since different concentrations were compared for each chemical, it is difficult to draw specific conclusions, but for the sensitizing chemicals both irritant and non-irritating sensitizing concentrations induced increases in expression. Additionally, *S100a8* expression in the skin peaked (all concentrations) at Day 7 and was signifi-cantly elevated for at least one concentration by Day 1 or 2 post-exposure. It is important to note that since the lungs were not perfused prior to collection, there is potential for contribution from the blood in the lung S100A8 response. This is reflected by their similar expression patterns. Neutrophils are known to express high levels of S100a8, and therefore circulating neutrophils could be contributing to the high levels of expression observed in multiple tissues. However, these finding suggest that differential expression patterns (early vs. later) could be a potential way to distinguish immunological mechanisms of disease.

The burden of occupational allergic disease is widespread. Occupational allergic conditions are multifactorial and are the result of complicated immunologic events. The results from the studies here suggest unique gene expression profiles are detectable following exposure to various antimicrobial chemicals, indicating potential utility as biomarkers in future risk assessment. Likewise, the data from these studies suggest a high throughput gene expression kinetics screen which can potentially serve as a basis for future investigational studies. The findings also support the need for additional research into mechanisms of disease, the mediators involved, and identification of potential biomarkers. Future studies will need to focus on additional earlier timepoints and evaluation of additional classes of chemicals. A complete understanding of the mechanisms of immune and allergic diseases resulting from LMW chemical exposure will allow for surveillance, proper treatment and/or prevention, while hazard identification will lead to risk assessment, which will ensure safe environments and effective exposure limits.

**Funding Information**

This work was supported by internal funds from the Health Effects Laboratory Division of the National Institute for Occupational Safety and Health.

**Declaration of interest**

The authors declare no conflicts of interest. The authors alone are responsible for the content of this manuscript. The findings and conclusions in this report are those of the authors and do not necessarily represent the views of the National Institute for Occupational Safety and Health, Centers for Disease Control and Prevention.

**References**

Anderson S, Franko J, Kashon M, Anderson K, Hubbs A, Lukomska E, Meade B. 2013. Expo-sure to triclosan augments the allergic response to ovalbumin in a mouse model of asthma. *Toxicol. Sci.* 132:96-106.

Anderson S, and Meade B. 2014. Potential health effects associated with dermal exposure to occupational chemicals. *Environ. Health Insights* 8:51-62.

Anderson S, Meade B, Long C, Lukomska E, Marshall N. 2016a. Investigations of immuno-toxicity and allergic potential induced by topical application of triclosan in mice. *J. Immunotoxicol.* 13:165-172.

Anderson S, Shane H, Long C, Lukomska E, Meade B, Marshal N. 2016b. Evaluation of the irritancy and hypersensitivity potential following topical application of didecyldimethylam-monium chloride. *J. Immunotoxicol.* 13:557-566.

Anderson S, Siegel P, Meade, B. 2011. The LLNA: A brief review of recent advances and limitations. *J. Allergy* 2011:424203.

Anderson S, Umbright C, Sellamuthu R, Fluharty K, Kashon M, Franko J, Jackson L, Johnson V, Joseph P. 2010. Irritancy and allergic responses induced by topical application of *o*-phthalaldehyde. *Toxicol. Sci.* 115:435-443.

Anderson S, Weatherly L, Shane H. 2019. Contribution of antimicrobials to the development of allergic disease. *Curr. Opin. Immunol.* 60:91-95.

Bangert C, Brunner P, Stingl G. 2011. Immune functions of the skin. *Clinics Dermatol.* 29:360-376.

Bello D, Herrick C, Smith T, Woskie S, Streicher R, Cullen M, Liu Y, Redlich C. 2007. Skin exposure to isocyanates: Reasons for concern. *Environ. Health Perspect.* 115:328-335.

Bernstein J, Stauder T, Bernstein D, Bernstein I. 1994. A combined respiratory and cutaneous hypersensitivity syndrome induced by work exposure to quaternary amines. *J. Allergy Clin. Immunol.* 94:257-259.

Cashman M, Reutemann P, Ehrlich A. 2012. Contact dermatitis in the United States: Epidemiol-ogy, economic impact, and workplace prevention. *Dermatol. Clin.* 30:87-98.

Chipinda I, Hettick J, Siegel, P. 2011. Haptenation: Chemical reactivity and protein binding. *J. Allergy* 2011:839682.

Cosmi L, Liotta F, Maggi L, Annunziato F. 2017. Role of Type 2 innate lymphoid cells in allergic diseases. *Curr. Allergy Asthma Rep.* 17:66.

Dahlgren M, and Molofsky A. 2018. All along the watchtower: Group 2 innate lymphoid cells in allergic responses. *Curr. Opin. Immunol.* 54:13-19.

Dearman R, and Kimber I. 1991. Differential stimulation of immune function by respiratory and contact chemical allergens. *Immunology* 72:563-570.

Dejobert Y, Martin P, Piette F, Thomas P, Bergoend H. 1997. Contact dermatitis from didecyldi-methylammonium chloride and bis-(aminopropyl)-lauryl amine in a detergent-disinfectant used in hospital. *Contact Dermatitis* 37:95-96.

Dibo M, and Brasch J. 2001. Occupational allergic contact dermatitis from *N,N*-bis(3-amino-propyl)-dodecylamine and dimethyldidecylammonium chloride in two hospital staff. *Contact Dermatitis* 45:40.

Dotson G, Maier A, Siegel P, Anderson S, Green B, Stefaniak A, Codispoti C, Kimber I. 2015. Setting occupational exposure limits for chemical allergens - understanding the challenges. *J. Occup. Environ. Hyg.* 12(S1):S82-98.

Fang J, Stingley R, Beland F, Harrouk W, Lumpkins D, Howard P. 2010. Occurrence, efficacy, metabolism, and toxicity of triclosan. *Environ. Carcinogen. Ecotoxicol. Rev.* 28:147-171.

Fujita H. 2013. The role of IL-22 and T_H_22 cells in human skin diseases. *J. Dermatol. Sci.* 72:3-8.

Fujita H, Sawada Y, Ogawa M, Endo Y. 2007. Health hazards from exposure to *o*-phthalalde-hyde, a disinfectant for endoscopes, and preventive measures for health care workers. *J. Occup. Health* 49:1-8.

Glaser A. 2004. The ubiquitous triclosan: A common antibacterial agent exposed. *Pesticides and You* 24:12-17.

Gonzalez M, Jegu J, Kopferschmitt M, Donnay C, Hedelin G, Matzinger F, Velten M, Guilloux L, Cantineau A, de Blay F. 2014. Asthma among workers in healthcare settings: role of disinfection with quaternary ammonium compounds. *Clin. Exp. Allergy* 44:393-406.

Herrick C, Xu L, McKenzie A, Tigelaar R, Bottomly K. 2003. IL-13 is necessary, not simply sufficient, for epicutaneously-induced T_H_2 responses to soluble protein antigen. *J. Immunol.* 170:2488-2495.

Isaac J, and Scheinman P. 2017. Benzalkonium chloride: An irritant and sensitizer. Dermatitis: 28:346-352.

Jia L, and Wu C. 2014. The biology and functions of T_H_22 cells. *Adv. Exp. Med. Biol.* 841:209-230.

Kim B, Siracusa M, Saenz S, Noti M, Monticelli L, Sonnenberg G, Hepworth M, van Voorhees, A, Comeau M, Artis D. 2013. TSLP elicits IL-33-independent innate lymphoid cell responses to promote skin inflammation. *Sci. Transl. Med*. 5:170ra116.

Kimber I, and Dearman R. 1992. The mechanisms and evaluation of chemically-induced allergy. *Toxicol. Lett.* 64-65:79-84.

Kohler J, Martin S, Pflugfelder U, Ruh H, Vollmer J, Weltzien H. 1995. Cross-reactive trinitro-phenylated peptides as antigens for Class II major histocompatibility complex-restricted T-cells and inducers of contact sensitivity in mice. Limited T-cell receptor repertoire. *Eur. J. Immunol.* 25:92-101.

Landsteiner K, and Jacobs J. 1935. Studies on the sensitization of animals with simple chemical compounds. *J. Exp. Med.* 61:643-656.

Long C, Marshall N, Lukomska E, Kashon M, Meade B, Shane H, Anderson S. 2016. A role for regulatory T-cells in a murine model of epicutaneous toluene diisocyanate sensitization. *Toxicol. Sci.* 152:85-98.

Manetz T, and Meade B. 1999. Development of a combined irritancy/phenotypic analysis assay for the identification and differentiation of chemicals with the potential to elicit irritation, IgE-mediated, or T-cell-mediated hypersensitivity responses. *Am. J. Indust. Med.* S1:136-138.

Marshall N, Lukomska E, Long C, Kashon M, Sharpnack D, Nayak A, Anderson K, Meade B, Anderson S. 2015. Triclosan induces thymic stromal lymphopoietin in skin promoting T_H_2 allergic responses. *Toxicol. Sci.* 147:127-139.

Marshall N, Lukomska E, Nayak A, Long C, Hettick J, Anderson S. 2017. Topical application of anti-microbial chemical triclosan induces immunomodulatory responses through the S100A8/A9-TLR4 pathway. *J. Immunotoxicol.* 14:50-59.

Mirshafiey A, Simhag A, El Rouby N, Azizi G. 2015. T_H_22 cells as a new player in chronic inflammatory skin disorders. *Intl. J. Dermatol.* 54:880-888.

Mowitz M, and Ponten A. 2015. Foot dermatitis caused by didecyldimethylammonium chloride in a shoe refresher spray. *Contact Dermatitis.* 73(6):374-6

Nayak A, Hettick J, Siegel P, Anderson S, Long C, Green B, Beezhold D. 2014. Toluene diiso-cyanate (TDI) disposition and co-localization of immune cells in hair follicles. *Toxicol. Sci.* 140:327-337.

Pralong J, Cartier A, Vandenplas O, Labrecque M. 2012. Occupational asthma: New low-molec-ular-weight causal agents, 2000-2010. *J. Allergy* 2012:597306.

Redlich C. 2010. Skin exposure and asthma: Is there a connection? *Proc. Am. Thor. Soc.* 7:134-137.

Ruiz Oropeza A, Fischer Friis U, Duus Johansen J. 2011. Occupational contact urticaria caused by didecyl dimethyl ammonium chloride. *Contact Dermatitis* 64:297-298.

Salimi M, Barlow J, Saunders S, Xue L, Gutowska-Owsiak D, Wang X, Huang L, Johnson D, Scanlon S, McKenzie A, et al. 2013. A role for IL-25 and IL-33-driven Type-2 innate lymphoid cells in atopic dermatitis. *J. Exp. Med.* 210:2939-2950.

Sasseville D. 2012. Acrylates in contact dermatitis. *Dermatitis.* 23:6-16.

Savage J, Johns C, Hauser R, Litonjua A. 2014. Urinary triclosan levels and recent asthma exacerbations. *Ann. Allergy Asthma Immunol.* 112:179-181.

Savage J, Matsui E, Wood R, Keet C. 2012. Urinary levels of triclosan and parabens are associated with aeroallergen and food sensitization. *J .Allergy Clin. Immunol.* 130:453-460.

Shaffer M, and Belsito D. 2000. Allergic contact dermatitis from glutaraldehyde in health-care workers. *Contact Dermatitis* 43:150-156.

Shane H, Long C, Anderson S. 2019a. Novel cutaneous mediators of chemical allergy. *J. Immunotoxicology* (in press).

Shane H, Lukomska E, Kashon M, Anderson S. 2019b. Topical application of the quaternary ammonium compound didecyldimethylammonium chloride activates Type 2 innate lymphoid cells and initiates a mixed-type allergic response. *Toxicol. Sci.* 168:508-518.

Shane H, Lukomska E, Stefaniak A, Anderson S. 2017. Divergent hypersensitivity responses following topical application of the quaternary ammonium compound, didecyldimethyl-ammonium bromide. *J. Immunotoxicol.* 14:204-214.

Shutty B, and Scheinman P. 2017. Occupationally-induced allergic contact dermatitis to aero-solized quaternary ammonium compounds. *Dermatitis*. 28:369.

Skaliy P, Thompson T, Gorman G, Morris G, McEachern H, Mackel D. 1980. Laboratory studies of disinfectants against *Legionella pneumophila*. *Appl. Environ. Microbiol.* 40:697-700.

Sokol W. 2004. Nine episodes of anaphylaxis following cystoscopy caused by Cidex OPA (*o*-phthalaldehyde) high-level disinfectant in four patients after cytoscopy. *J. Allergy Clin. Immunol.* 114:392-397.

Suneja T, and Belsito D. 2008. Occupational dermatoses in healthcare workers evaluated for suspected allergic contact dermatitis. *Contact Dermatitis* 58:285-290.

Weatherly L, and Gosse J. 2017. Triclosan exposure, transformation, and human health effects. *J. Toxicol. Environ. Health* 20:447-469.

Zhang X, Murray D, Lewis D, Siegel P. 2002. Dose-response and time course of specific IgE and IgG after single and repeated topical skin exposure to dry trimellitic anhydride powder in a Brown Norway rat model. *Allergy* 57:620-626.

Zissler U, Esser-von Bieren J, Jakwerth C, Chaker A, Schmidt-Weber C. 2016. Current and future biomarkers in allergic asthma. *Allergy* 71:475-494.

**Figure legends**

**Figure 1.** Increases in mRNA expression of danger signals following antimicrobial chemical exposure in the mouse skin. Fold-change in expression of **(A)** *Tslp* and **(B)** *S100a8* following 1, 2, 4, and 7 days on exposure. Points represent mean (± SEM) of five mice/group. Low, mid and high concentration of each chemical were evaluated. Statistical significance (p < 0.05) compared to 0% is indicated at each timepoint for *low, @mid, and ^#^high concentrations for each chemical. Dotted line represents an arbitrary value for baseline fold-change.

**Figure 2.** Increases in mRNA expression of *S100a8* in the **(A)** blood (A) and lung (B) following antimicrobial chemical exposure on the mouse skin following 1, 2, 4, or 7 days of exposure. Points represent mean (± SEM) of five mice/group. Low, mid and high concentration of each chemical were evaluated. Statistical significance (p < 0.05) compared to 0% is indicated at each timepoint for *low, @mid, and ^#^high concentrations for each chemical. Dotted line represents an arbitrary value for baseline fold-change.

**Figure 3**. Increases in mRNA expression of *Il4* following antimicrobial chemical exposure on the mouse skin. Fold-change in expression of *Il4* in the **(A)** skin and **(B)** dLN following 1, 2, 4, and 7 days of exposure. Points represent mean (± SEM) of five mice/group. Low, mid and high concentration of each chemical were evaluated. Statistical significance (p < 0.05) compared to 0% is indicated at each timepoint for *low, @mid, and ^#^high concentrations for each chemical. Dotted line represents an arbitrary value for baseline fold-change.

**Figure 4.** Increases in mRNA expression of T_H_2 cytokines in lungs following antimicrobial chemical exposure on mouse skin. Fold-change in expression of **(A)** *Il4* and **(B)** *Il13* in the mouse lung following 1, 2, 4, and 7 days of exposure. Points represent mean (± SEM) of five mice/group. Low, mid and high concentrations were evaluated. Statistical significance (p < 0.05) compared to 0% is indicated at each timepoint for *low, @mid, and ^#^high concentrations. Dotted line represents an arbitrary value for baseline fold-change.

**Figure 5.** Increases in mRNA expression of regulatory genes and T_H_1 cytokines following anti-microbial chemical exposure in the mouse skin following 1, 2, 4, and 7 days of exposure. Fold-change in the expression of **(A)** *Foxp3* in skin and **(B)** *Il10* in dLN. Points represent mean (± SEM) of five mice/group. Low, mid and high concentration of each chemical were evaluated. Statistical significance (p < 0.05) compared to 0% is indicated at each timepoint for *low, @mid, and ^#^high concentrations for each chemical. Dotted line represents an arbitrary value for baseline fold-change.

**Figure 6**. Unique changes in mRNA expression of genes following antimicrobial exposure. Fold-change in expression of **(A)** *Tlr4* and **(B)** *Cdh1* in the skin following 1, 2, 4 and 7 days of exposure. Points represent mean (± SEM) of five mice/group. Low, mid and high concentration of each chemical were evaluated. Statistical significance (p < 0.05) compared to 0% is indicated at each timepoint for *low, @mid, and ^#^high concentrations for each chemical.

**Figure 7.** Increase in mRNA expression of cytokines following chemical exposure. Fold-change in expression of **(A)** *Ifnγ* in dLN and **(B)** *Il22* in skin following 1, 2, 4, and 7 days of exposure. Points represent mean (± SEM) of five mice/group. Low, mid and high concentration of each chemical were evaluated. Statistical significance (p < 0.05) compared to 0% is indicated at each timepoint for *low, @mid, and ^#^high concentrations for each chemical. Dotted line represents an arbitrary value for baseline fold-change.

**Table 1.** **Classification of antimicrobial chemicals used in this study.**

| **Test Article** | **Test Concentrations** | **Uses** | **Disease Outcomes** | **Classification** |
| --- | --- | --- | --- | --- |
| Benzalkonium chloride (BAC) | 0.5, 1, and 2% | Surface Disinfectant | Skin irritation, asthma | Irritant and or T-cell mediated sensitizer  (Manetz and Meade, 1999; Isaac and Scheinman, 2017) |
| Didecyl dimethyl ammonium chloride (DDAC) | 0.125, 0.25 and *0.5%  (*irritating concentration) | Surface Disinfectant | Skin irritation, asthma, allergic contact dermatitis | T-cell-mediated sensitizer  (Anderson et al., 2016b) |
| *o*-phthaldialdehyde (OPA) | 0.25, 0.5, and *0.75%  (*irritating concentration) | Sterilization of medical devices | IgE-mediated hypersensitivity, allergic contact dermatitis | IgE mediated sensitizer  (Anderson et al., 2010) |
| Triclosan (TCS) | 0.75, 1.5, and 3% | Antimicrobial soap | Enhances severity and frequency of food and aeroallergy | Adjuvant  (Anderson et al., 2013, 2016a) |

Three concentrations were selected for each test chemical and defined as low, mid, and high.

**Supplemental figure legends**

**Supplemental Figure 1.** Expression of genes in blood following antimicrobial chemical exposure on mouse ears. Heat map of fold-change in expression compared to vehicle control. Each box represents mean of five mice/group. Low, mid, and high concentration of each chemical were evaluated. Data is not available for DDAC Day 7.

**Supplemental Figure 2.** Expression of genes in the ears following antimicrobial chemical exposure on mouse ears. Heat map of fold-change in expression compared to vehicle control. Any fold-changes > 50 are displayed in heat map as 50. Each box represents mean of five mice/group. Low, mid, and high concentration of each chemical were evaluated.

**Supplemental Figure 3.** Expression of genes in dLN following antimicrobial chemical exposure on mouse ears. Heat map of fold-change in expression compared to vehicle control. Any fold-changes > 10 are displayed in heat map as 10. Each box represents mean of five mice/group. Low, mid, and high concentration of each chemical were evaluated.

**Supplemental Figure 4.** Expression of genes in lung following antimicrobial chemical exposure on mouse ears. Heat map of fold-change in expression compared to vehicle control. Each box represents mean of five mice/group. Low, mid, and high concentration of each chemical were evaluated.

Supplemental Figure 1

Supplemental Figure 2

Supplemental Figure 3

Supplemental Figure 4
